# Supplementary material for: Chromothripsis during telomere crisis is independent of NHEJ, and consistent with a replicative origin
Source: Genome Res. 2019 May;29(5):737–49. doi: 10.1101/gr.240705.118 (PMC6499312; doi:10.1101/gr.240705.118)
Supplement: Supplemental Material [file supp_gr.240705.118_Supplemental_file_1.zip › contigs/annotated_contigs/DB38/contig.2.DB38_length_677_mean_cov_8.41949778434.docx]

**DB38_length_677_mean_cov_8.41949778434**

```
TGCCCTTGGTAGCCTCCCTCTGCTTTCACACAGTTCTATCTCCTTCTCTCTCAGCCTCACTTGAGTTCATTATCACAGCTCCTGTCAAG
 >chr5:145974069-145974427 - E=1e-200
GACTGAGGACCCACCAGAGCAAGCTGGAGGCAGGTTGCTAGTGCAGCTCAGAGTGTGCACTCCAGAGCCTGATACCGGGGCTCAGTTCC

CTCCCCTTCTGTCACTTTTTCTGTGTGATCTTGGACAAATGATGTCTTATCTCTGTGCTTCATGCTCCATTAAAGTAAAGAGAATGAAA

GTACCTACATCATAAGATGGCTTTAGAGATGAAATGAGGTTTTGACAGATAAAGCACCTGGCTAATAATGACAAGCCAGCATATTGGGT

AT|ACCAGTCCCCATCACTGCTCATGCCCTGGTCCAGGCCAGAATCTATCACTCAGGTTATAACTGGTCTCCCTCCCTTTGCCTTTGCA
    >chr5:145973216-145973535 - E=3e-180
CCCTTAGGATCCATTGCAACACAGCAGATTCAGCAATCCTTTCTGTGAGGTCTCTTCTTCCTCAGAATAAAGGGCCTATGTAAACTGAT

CATCCTCTCCCCCAGCTGCTTACTCACTCCTCTCCCACCACACTCCCTCCAGGATTCCTGCCTCTAGGCCTTTATGTTTGCTGTTTCCT

ATGCTTGGAGAGCCTTCCCCTCACCCCAAACCCAGACATCACTGTGGCTCATTCA
```
